# The Role of the Cutaneous Mycobiome in Atopic Dermatitis

**DOI:** 10.3390/jof8111153

**Published:** 2022-10-31

**Authors:** Milena Szczepańska, Leszek Blicharz, Joanna Nowaczyk, Karolina Makowska, Mohamad Goldust, Anna Waśkiel-Burnat, Joanna Czuwara, Zbigniew Samochocki, Lidia Rudnicka

**Affiliations:** 1 Department of Dermatology, Medial University of Warsaw, 02-008 Warsaw, Poland; 2 Department of Dermatology, University Medical Center, 55131 Mainz, Germany

**Keywords:** atopic dermatitis, *Candida*, dysbiosis, fungal microbiome, *Malassezia*, mycobiome

## Abstract

Atopic dermatitis is a chronic inflammatory skin disorder characterized by eczematous lesions, itch, and a significant deterioration in the quality of life. Recently, microbiome dysbiosis has been implicated in the pathogenesis of atopic dermatitis. Changes in the fungal microbiome (also termed mycobiome) appear to be an important factor influencing the clinical picture of this entity. This review summarizes the available insights into the role of the cutaneous mycobiome in atopic dermatitis and the new research possibilities in this field. The prevalence and characteristics of key fungal species, the most important pathogenesis pathways, as well as classic and emerging therapies of fungal dysbiosis and infections complicating atopic dermatitis, are presented.

## 1. Introduction

Atopic dermatitis (AD) is a chronic, relapsing, inflammatory dermatosis characterized by eczema, itch, and a frequent association with other atopic disorders [1]. AD is diagnosed worldwide with a prevalence of up to 20.1% in children and 4.9% in adults [2]. It is associated with a significant deterioration in the quality of life, constituting a considerable burden for patients and a challenge for healthcare systems [3].

The pathogenesis of AD is multifactorial and not entirely elucidated. It has been suggested that genetic predisposition, skin barrier malfunction, and disordered innate and acquired immune responses have the greatest impact on the development of AD [1]. These processes trigger dysbiosis of the cutaneous microbiome, which further aggravates skin barrier damage and immune imbalances and increases the risk of secondary infections, complicating the course of AD [4].

Since Leyden et al. first reported the prominent colonization of AD lesions by *Staphylococcus aureus*, most studies have focused on the analysis of the bacterial microbiome [5,6]. It was shown that *S. aureus* causes displacement of other bacterial species, which disrupts the cutaneous homeostasis dependent on host-microbiome signaling [7]. Numerous virulence factors of *S. aureus*, such as enterotoxins, phenol-soluble modulins, hemolysins, and exogenous proteases, have been reported to induce Th2-type inflammation and damage the epidermal barrier, thereby aggravating the course of AD [8].

The relative paucity of data regarding the role of the fungal microbiome (also called the mycobiome) in AD was partly associated with methodological challenges. However, recent advances in high-throughput sequencing have provided new insights into the cutaneous fungal communities in AD by complementing the data from culture-based studies. The aim of this review is to summarize the current knowledge of the role of the cutaneous mycobiome in AD, as well as discuss the clinical implications and new opportunities for research in this field.

## 2. Materials and Methods

A literature search was performed on the following databases: PubMed, Scopus, and Web of Science. The keywords used to perform the search were “atopic dermatitis”, “fungi”, “fungal microbiome”, “mycobiome”, “*Candida*”, “*Malassezia*”, and “yeasts” in different combinations. Only articles in English were included. 

## 3. Methods of Identifying the Cutaneous Mycobiome

Microbial communities can be studied using culture-based or molecular approaches [9]. Although the culture-based identification of fungi remains the gold standard in clinical practice, it is also associated with high time consumption, limited ability to investigate a wide range of microbes in selected conditions, and uncertainty in discriminating between different species due to the mainly phenotypic analyses of the obtained colonies. The sensitivity of culture-based methods is generally lower than that of molecular studies, which may be associated with the difficulty in obtaining positive cultures, particularly in species with high growth requirements. 

The development of molecular biology has aided mycobiome studies by enabling rapid and reliable investigations of fungi using techniques such as polymerase chain reaction (PCR) and restriction fragment length polymorphism [10]. Numerous loci have been mapped to provide a targeted identification of different taxa. For example, to identify the *Malassezia* genus, the D1/D2 region of 26S rDNA is frequently used [11]. Genes encoding β-tubulin, chitin synthase 2, and polymerase II large subunit are also implemented in studies of fungal taxonomy [12,13,14,15]. Importantly, most of these methods are applied to the fungal isolate and not directly to the biological product. 

**Figure 1:**
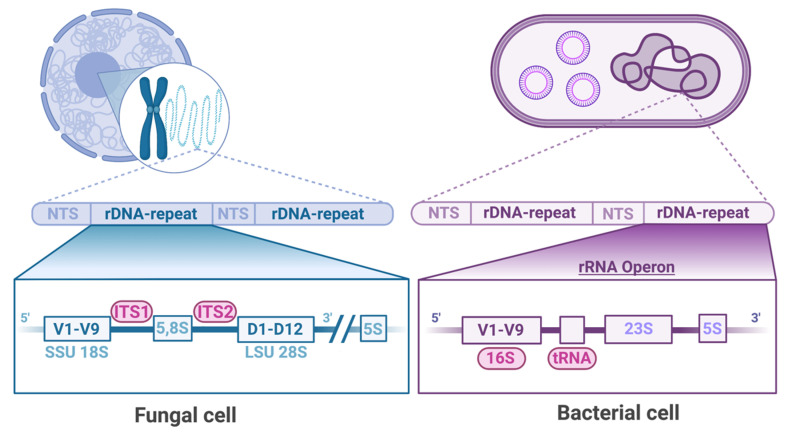
Comparison of targets for next-generation sequencing studies of fungal and bacterial microbiomes. The fungal microbiome is primarily identified by sequencing the internal transcribed spacer (ITS) fragments. The bacterial microbiome is analyzed by sequencing the prokaryotic 16S rRNA subunit or tRNA. rDNA—reverse DNA; ITS—internal transcribed spacer; LSU—large ribosomal subunit; tRNA—transfer RNA; NTS—non-transcribed spacer; SSU—small ribosomal subunit.

The comprehensive identification of the microbial communities present in numerous microniches in the human body has become possible thanks to the next-generation sequencing of conservative prokaryotic and eukaryotic genome fragments. Although the bacterial microbiome is most frequently analyzed by sequencing the prokaryotic 16S rRNA subunit, the mycobiome can be identified by sequencing primarily the ITS fragments [16,17] (Figure 1). The latter have been universally accepted as the most suitable for mycobiome analysis, especially using the updated ITS2 and ITS1F primers [18,19].

ITS amplicon sequencing is a reliable and relatively quick method of analyzing the mycobiome even in low biomass samples, which is typical in the case of harvesting material from the skin. However, it has limited discriminative properties and does not differentiate between live or dead microorganisms. Metagenomic or metatranscriptomic approaches can be used to provide more insights into the microbiota [20]. To date, few studies have implemented those methods in AD.

To identify the metabolic pathways of microorganisms and better understand the molecular interactions with the host, whole-genome sequencing can be used. Among others, whole-genome sequencing has enabled the identification of similar gene families encoding hydrolases and lipases in the opportunistic species *Malassezia globosa* and *Candida albicans*, which points to the role of those genes in both asymptomatic skin colonization and opportunistic infection [21].

Despite the apparent superiority of the sequencing approach, fungal communities may be even more fully appreciated by using a combination of culture and sequencing. This methodology is relatively common and was used, among others, by Hamm et al. to help in discriminating the seasonal variation and microhabitat preference of keratinophilic fungi [22]. Although the sequencing approach enables the identification of a wider spectrum of non-culturable fungi, cultures highlight the relative potential of different fungal species to proliferate in favorable conditions. Furthermore, the testing of susceptibility to antifungal agents and the pathogenicity of fungi also require culture-based methods [23,24]. In this regard, future studies of mycobiome in AD could provide more insights if a combination of those two techniques is used.

## 4. Mycobiome in Normal Skin

Skin microbiota plays an important role in immune homeostasis, maturation of the epidermis, and protection against pathogens [25]. Commensal microorganisms occupy niches with a microenvironment supporting their growth. Therefore, certain locations (e.g., moist, dry, and sebaceous) tend to harbor microbial communities that are comparable between different individuals [26]. Nevertheless, the composition of the ‘physiological’ microbiota can be influenced by several factors such as age, ethnicity, hygienic habits, temperature, and moisture [27,28,29]. Therefore, an adequate interpretation of mycobiome studies in patients with AD requires the knowledge of its composition and the variables that affect healthy individuals.

Initial culture-dependent studies of the cutaneous mycobiome provided data on the most abundant cultivable fungal taxa [27,29,30,31]. The genera *Malassezia*, *Rhodotorula*, *Debaryomyces*, *Cryptococcus*, and occasionally *Candida* were identified as fungal skin commensals. A more recent study comparing the results of culture-based and sequencing approaches in fourteen skin sites identified >130 cultivable isolates belonging to the genera *Malassezia*, *Penicillium*, *Aspergillus*, *Alternaria*, *Candida*, *Chaetomium*, *Chrysosporium*, *Cladosporium*, *Mucor*, *Rhodotorula*, and *Trichophyton* [18].

Amplicon sequencing revealed the presence of a wider spectrum of cutaneous fungal communities belonging primarily to the phyla *Ascomycota* (91.22% of total fungal species, of which 323 were identified as human pathogens; examples of genera of this phylum are *Aspergillus*, *Candida*, *Coccidioides*, *Fusarium*, and *Pneumocystis*) and Basidiomycota (4.34% of total fungal species, of which 43 were identified human pathogens; examples of genera of this phylum are *Malassezia*, *Cryptococcus*, and *Trichosporon*) [18,32].

The most prevalent species at all core body and arm sites belonged to the *Malassezia* genus [18,29,33]. Their abundance was the highest in sebaceous areas. This is because *Malassezia* spp. lack the fatty acid synthase, rendering them dependent on the breakdown of exogenous fats for their own lipid synthesis [34]. *M. restricta* predominated in the external auditory canal, retroauricular crease, and glabella, whereas *M. globosa* was found predominantly on the back, occiput, and inguinal crease. The antecubital crease, volar forearm, hypothenar palm, and nares were characterized by a greater diversity of the *Malassezia* genus (*M. restricta*, *M. globosa*, and *M. sympodialis*) [18,27]. The feet displayed a significantly more diverse mycobiome than other locations, involving the genera *Malassezia*, *Aspergillus*, *Cryptococcus*, *Rhodotorula*, *Epicoccum*, and others [9,18]. The presence of a more heterogenous array of fungi on the feet could be associated with several factors. Firstly, the expression of sebaceous glands is lower than in other locations such as the head and neck area [26]. Hence, the mycobiome of the feet is not dominated by lipophilic yeasts. Secondly, the use of footwear creates a humid and warm microenvironment favoring the development of a wide range of fungal species [35]. Finally, the unavoidable exposition of the foot to the outer environment favors the acquisition of diverse fungi. Indeed, species belonging to the genera *Cryptococcus*, *Rhodotorula*, *Aspergillus*, and *Epicoccum* are frequently present in the soil, air, water, and vegetation [36,37,38]. The plantar heel was characterized by the greatest fungal diversity (median richness of approx. 80 genera), followed by other parts of the foot (i.e., the toeweb and toenail were characterized by a median richness of approx. 60 and 40 genera, respectively) [18].

One study implementing a combination of culture-based and molecular analyses of a cohort from Switzerland and Singapore showed significant ethnic and geographical differences in mycobiome composition [29]. Singaporean subjects were colonized by a higher number of *Malassezia* species than Swiss subjects (2.03 species vs. 1.55 species, *p* < 0.05), highlighting the effect of temperature and moisture on mycobiota composition. Additionally, distinct clusters were distinguished for both Caucasian individuals in Singapore and Switzerland and different groups in Singaporean society, which suggests the presence of ethnicity-dependent factors affecting the fungal communities of the skin.

Differences in cutaneous mycobiome composition were also reported to depend on sex and age. For example, the mycobiome of the forehead and cheeks of Korean women aged 60–63 years has been shown to display higher alpha diversity than those aged 19–28 years [39]. In another report, women aged 19–29 years were characterized by a lower abundance of *Malassezia* species on the skin than men of the same age and women of other ages. Nevertheless, the authors concluded that this observation might have been biased by the more frequent use of cosmetics in young women [40]. It was also demonstrated that the prevalence of *Malassezia* on the skin increases during pregnancy and several months after giving birth, which underlies the probable influence of hormonal changes on the activity of cutaneous exocrine glands [41].

Children display even more prominent differences in the fungal microbiota. In the first month of life, *Candida* was reported to be the dominating genus and *Malassezia* accounted for only 2% of the skin fungal communities [42]. The most frequently isolated species included *C. tropicalis*, *C. parapsilosis*, *Saccharomyces cerevisiae*, *C. albicans*, and *C. orthopsilosis*. Another study showed that the proportions of Ascomycota and Basidiomycota were comparable throughout the first 6 months of life. The Ascomycota phylum was represented mainly by *Alternaria* (10.73%), *Cladosporium* (7.00%), *Candida* (5.91%), and *Aspergillus* (2.46%), and the Basidiomycota phylum by *Malassezia* (36.43%) and *Cryptococcus* (3.21%) [43]. Among the genus *Malassezia*, *M. globosa* was the most represented species (48.17%), whereas others were identified with lower frequencies: *M. restricta* (29.45%), *M. furfur* (6.85%), *M. obtusa* (3.6%), *M. sympodialis* (1.23%), and *M. japonica* (0.3%) [43]. Furthermore, Jo et al. compared the cutaneous mycobiomes of older children (<14 years of age) and adults [44]. The children were shown to harbor a higher diversity of Ascomycota and a lower relative abundance of *Malassezia. Aspergillus*, *Epicoccum*, and *Phoma* constituted over 5% of the identified genera in 40.2% of samples from children in comparison to only 9.5% of samples from adults [44]. 

## 5. Cutaneous Mycobiome in Patients with Atopic Dermatitis

AD skin is characterized by reduced lipid content, higher pH, and increased transepidermal water loss [45,46,47,48]. These phenomena cause changes in the propensity for fungal growth, thereby affecting the results of mycobiome assays. 

Initial culture-based analyses of the AD mycobiome focused on easily cultivable yeasts. The genus *Malassezia* was studied extensively (Table 1). Most reports revealed similar or lower rates of *Malassezia* colonization in patients with AD than in healthy controls [49,50,51,52,53]. Additionally, *Malassezia* species were isolated less frequently from the lesional skin than from the nonlesional skin of AD individuals [51]. In line with these findings, semi-quantitative cultures revealed a lower abundance of *Malassezia* on the lesional and nonlesional skin of patients with AD than on the skin of healthy controls [49,51]. *Malassezia* abundance in AD patients was comparable in all analyzed locations, i.e., the scalp, forehead, trunk, arms, and legs, whereas in healthy subjects, it was considerably lower on the arms and legs than in other microniches. On the species level, *M. sympodialis*, *M. globosa*, and *M. furfur* were identified most frequently using the culture-based approach [49,50,51,54,55].

In most of the above studies, the culture results were verified with PCR-based techniques, whereas others implemented the latter as the basic methodology [11,50,56,57]. The findings were mostly concurrent with the phenotypic identification of the cultivated yeasts. However, in contrast to the cultures, nested PCR showed that *M. globosa* and *M. restricta* were more abundant on AD skin than *M. sympodialis* [11,56,58]. These differences could be explained by the fact that among the *Malassezia* spp., *M. restricta* and *M. globosa* have the most fastidious growth conditions and therefore might be underrepresented in studies using solely the culture-based approach [59]. The mean number of *Malassezia* species in patients with AD was higher than in healthy controls (2.7 species vs. 1.8 species detected per individual, respectively, in a study by Sugita et al. [11]; 4.1 ± 1.9 vs. 2.8 ± 0.8 species per individual, respectively, in a study by Tajima et al. [56]).

A few culture-based studies analyzed the carriage of other yeasts in patients with AD and their results were often contradictory. Arzumanyan et al. reported that both the lesional and nonlesional skin of AD patients were colonized more frequently by *Candida* than the skin of the healthy population [60], whereas Javad et al. did not report such differences [61]. Another report did not identify any AD patients with skin cultures positive for *C. albicans*, whereas 59% of individuals exhibited the carriage of this yeast in the nasopharynx [62]. A PCR-based study revealed that patients with AD showed higher rates of colonization by *Candida* spp. than healthy controls, with *C. albicans* and *C. parapsilosis* being the most common isolates [58]. Reports implementing cultures with subsequent DNA sequence analysis of ITS and the D1/D2 26S rRNA gene revealed a more frequent carriage of *Cryptococcus diffluens*, *C. liquefaciens*, and *C. albidus* on the skin of patients with AD than in healthy individuals [63,64]. 

Sequencing studies provided further insights into the role of the mycobiome in AD. Zhang et al. identified the *Malassezia* genus as the most abundant on AD skin [65]. *M. globosa* and *M. restricta* were the most abundant species. In patients with mild to moderate AD, *M. restricta* dominated over *M. globosa* but in severe cases, this ratio was close to one. This observation suggests that the level of epithelial barrier disruption correlating with the progressive depletion of cutaneous lipids affects the profile of *Malassezia* spp. colonizing the skin. Indeed, whole-genome sequencing and subsequent proteomic analysis of *M. globosa* and *M. restricta* revealed particularly high lipase activity in *M. globosa*, suggesting its better adaptation to the lipid-deficient skin of severe AD patients [66]. The diversity of non-*Malassezia* yeast microbiota (genus *Candida*, *Cryptococcus*, *Trichosporon*, and *Rhodotorula*) was higher in patients than in controls (13.0 ± 3.0 and 8.0 ± 1.9 species per individual, respectively), whereas filamentous fungi were retrieved at similar frequencies in patients and controls (5.2 ± 0.8 and 4.3 ± 0.8 species per individual, respectively). At the species level, the authors confirmed the above-mentioned tendency for colonization by *Cryptococcus diffluens* and *C. liquefaciens* in the AD group.

Concurrent results were reported by Schmid et al. *M. restricta* and *M. globosa* showed the highest relative abundance in AD, albeit with a lesser abundance of *M. restricta* in severe cases [67]. AD severity was also correlated with a lower relative abundance of Malasseziomycetes and higher diversity of other fungal taxa (mainly Saccharomycetes, with genus *Candida* and *Debaryomyces* predominating). Taken together, those findings suggest that the composition of the fungal microbiota of the skin correlates with AD severity, although it is not possible to determine whether mycobiome dysbiosis plays a causative role or is just an epiphenomenon secondary to epidermal barrier damage and cutaneous inflammation [65]. 

Han et al. compared the cutaneous mycobiomes of Korean patients with AD and healthy subjects [68]. The genus *Malassezia* was the most prevalent in both groups, but patients with AD showed a considerably higher inter-subject mycobiome variability and a tendency for higher differentiation of the fungal communities than healthy controls. At the species level, *M. globosa* and *M. restricta* were once again identified as the most abundant species in both AD and healthy individuals. *M. japonica* was shown to be an AD-dominant species and *M. slooffiae*, *M. obtusa*, and *M. yamatoensis* were detected only in the AD group. The correlation between mycobiome composition and AD severity was not assessed. 

In a targeted sequencing-based analysis of cutaneous eukaryotes, AD patients harbored a more diverse microbiota than healthy controls [69]. Among the fungi, Malasseziacae were the most abundant in both patients and healthy subjects. At the species level, *M. globosa* and *M. restricta* prevailed. Furthermore, *Geotrichum candidum* was significantly more common on both AD lesional and nonlesional skin (36% and 35% of samples, respectively) compared to healthy control skin (4% of samples). Species richness and general community composition were not correlated to disease severity and filaggrin mutations. 

Moosbrugger-Martinz et al. performed a comprehensive microbiome analysis of the popliteal fossa and scapular regions in patients with AD, Netherton syndrome, ichthyosis vulgaris, and healthy controls [70]. The mycobiome in AD showed a significant expansion of Ascomycota, which was correlated with serum IgE levels. In comparison with healthy individuals, the mycobiome of the popliteal fossa in AD patients was characterized by an increase in the class Dothideomycetes and the affiliated genus *Cladosporium*, which correlated with transepidermal water loss. The scapular region of AD patients demonstrated a reduction in *Malassezia* with a simultaneous expansion of *Cladosporium*, *Leptosphaeria*, and *Debaryomyces*. Interestingly, the overgrowth of *S. aureus* and *Cladosporium* was positively correlated in the popliteal fossa of patients with AD, highlighting the possible role of fungi in supporting the dysbiosis of the bacterial microbiota. 

Chng et al. performed a metagenomic study comparing cutaneous microbiomes in patients with AD and healthy controls. The eukaryotes were dominated by Malasseziaceae, with *M. globosa* and *M. restricta* reported to be the most frequently affiliated species [71]. Overall, *Malassezia*, including *M. globosa*, showed a significant depletion in AD. However, *M. dermatis* and *M. sympodialis* were characterized by a higher relative abundance in the AD group than in the controls. Aspergillaceae, the next most common eukaryotic member of the skin microbiota, was not depleted in AD individuals. Other metagenomic investigations failed to identify the presence of fungal communities on AD skin. In a healthy population, Nath et al. reported *M. globosa* in 8,89% of samples [72], whereas Bjerre et al. discovered it in relative abundance, ranging from 0.9 to 2.1% on antecubital flexures and from 0.1 to 3.4% on the neck [73].

### Cutaneous Mycobiome in Head and Neck Dermatitis

The head and neck are the often affected and treatment-recalcitrant regions in patients with AD [74]. The predominant involvement of these locations has been distinguished as the head and neck dermatitis variant of AD. As the head and neck are sebaceous locations, a hypothesis on the pathogenic role of *Malassezia* spp. in this entity has been raised [75]. Although most studies cited above involved the analysis of these regions, some reports focused specifically on the mycobiome composition of this form of AD.

Kaga et al. performed a nested PCR analysis of *Malassezia* colonization in patients with mild, moderate, and severe head and neck dermatitis and healthy controls [76]. The abundance of *Malassezia* in severe AD patients was two- to fivefold higher than in other groups. *M. globosa* and *M. restricta* were present in 80% of samples from AD patients but their relative abundance differed with disease severity. In mild and moderate patients, *M. restricta* predominated over *M. globosa* but in severe cases, the abundance of these species was almost identical. The diversity of *Malassezia* spp. in patients and controls was comparable (3.5–4.2 species per individual).

An analogous study showed comparable rates of the carriage of different *Malassezia* species in both patients with head and neck dermatitis and healthy individuals [77]. *M. globosa* and *M. restricta* were present in all samples collected from both groups. Patients with mild, moderate, and severe head and neck dermatitis showed similar diversity of *Malassezia* species (3.7 ± 1.6, 3.7 ± 1.6, and 3.5 ± 1.4 per individual, respectively). The number of identified species correlated with the total IgE antibody levels against *Malassezia*.

One report implemented ITS amplicon sequencing to characterize scalp mycobiome compositions in patients with AD and healthy controls [78]. Basidiomycota constituted a vast majority of the fungal communities. In both groups, *Malassezia* was the most abundant of the 71 identified genera. At the species level, *M. restricta* and *M. globosa* dominated. There was no correlation between mycobiome composition and patients’ age, gender, and disease severity. *M. restricta* showed a tendency to prevail over *M. globosa* in the AD group but the results were not statistically significant.

## 6. The Role of the Cutaneous Mycobiome in Atopic Dermatitis: Possible Pathways

To date, the exact mechanisms by which fungal dysbiosis influences the course of AD have not been entirely explained. The results of mycobiome assays reporting alterations primarily in the genus *Malassezia*, as well as the association of fungal dysbiosis with AD severity and head and neck dermatitis, have provided a basis for other investigations aiming to explain these phenomena.

### 6.1. Malassezia

**Figure 2:**
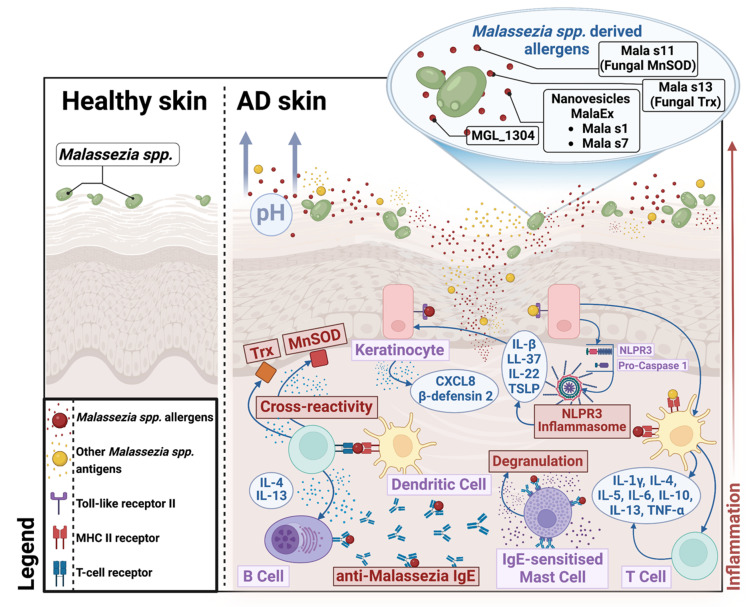
Proposed pathways of *Malassezia* spp. pathogenicity in atopic dermatitis. The skin of patients with atopic dermatitis is characterized by epidermal barrier damage, increased skin pH, and reduced lipid content, which facilitates the penetration of allergens and other antigens of *Malassezia* spp. These are recognized by antigen-presenting cells, such as keratinocytes and dendritic cells, inducing the activation of innate and acquired immune responses. This results in the upregulation of antimicrobial peptides; secretion of Th1, Th2, and Th17 cytokines; and production of specific IgE. Cross-reactivity of *Malassezia* allergens with human thioredoxin and manganese-dependent superoxide dismutase, as well as degranulation of specific IgE-sensitized mast cells, further contribute to the robust cutaneous inflammation. CXCL8—CXC motif chemokine ligand 8; MHC II receptor—major histocompatibility class II receptor; MnSOD—manganese-dependent superoxide dismutase; Trx—thioredoxin; TSLP—thymic stromal lymphopoietin.

The data presented in the previous chapters suggest that the abundance of *Malassezia* spp. is lower in patients with AD than in healthy subjects, which most probably results from the depletion of cutaneous lipids [65,70]. Despite that fact, *Malassezia* spp. seem to influence the course of AD, which may result from the combination of epithelial barrier damage, abnormal signaling via toll-like receptors, and disordered acquired immune responses [79]. Importantly, the presence of *Malassezia* spp. was found to induce a wide range of proinflammatory cytokines. The triggering of IL-17 and IL-23 was reported, particularly in a model of impaired epidermal barrier function [80]. Simultaneously, the internalization of *Malassezia* yeasts by monocyte-derived dendritic cells induced their maturation and Th2 cytokine secretion [81]. Based on the observations regarding the increased secretion of Th1-attracting CXC chemokine ligand 10 and STAT1 activity in human keratinocytes by *M. restricta*, the upregulation of the Th1 response was also implicated [82]. In vitro data further suggest that antigen-presenting cells and keratinocytes exposed to *Malassezia* activate the NLPR3 inflammasome, which results in the secretion of IL-1β, thymic stromal lymphopoietin, and antimicrobial peptides such as LL-37 and β-defensin 2 [83,84]. A higher expression of LL-37 upon exposition to *M. sympodialis* was confirmed in monocyte-derived dendritic cells of severe AD patients than in individuals with milder disease and healthy controls [85]. *M. sympodialis* extracts were further demonstrated to activate both nonsensitized and IgE-sensitized mast cells to release inflammatory mediators and modify IL-6 production [86]. Taken together, these data suggest a considerable dysregulation of the innate and acquired immune responses elicited by *Malassezia* in the presence of an epidermal barrier defect, which is typical for AD (Figure 2). 

The mechanistic studies cited above analyzed *Malassezia* spp. in their yeast form and not their hyphal form. It should be emphasized that this methodological assumption is probably correct since the hyphal form is seen primarily in pityriasis versicolor and some cases of seborrheic dermatitis but not in head and neck dermatitis [87].

#### 6.1.1. Malassezia-Derived Allergens

A relatively high proportion of AD patients presented reactivity to *Malassezia* allergens, as confirmed by skin prick tests, atopy patch tests, and increased specific IgE levels, which was not seen in healthy individuals [53,88]. The detection of specific IgE against *Malassezia* antigens in the serum of patients with AD was therefore thought to reflect their role in cutaneous inflammation and increased disease severity [30,63,65,89]. Traditionally, the immunogenic capability of *Malassezia* was attributed to cell wall lipids [30]. Recently, the role of other *Malassezia*-derived allergens has been proposed. 

Allergens associated with *Malassezia* show the ability to stimulate keratinocytes, monocytes, and dendritic cells [79,89,90,91]. This leads to an increase in Th2 cytokine secretion and the production of specific IgE. *Malassezia* spp. allergens, such as Mala s 11 and s 13 (encoding thioredoxin and manganese-dependent superoxide dismutase, respectively), demonstrate great similarities to the corresponding human enzymes triggering the cross-reactivity of CD4+ T lymphocytes and subsequent induction of Th2/Th17-dependent skin inflammation [92]. Another allergen derived from *M. globosa*, MGL_1304, was identified in sweat [93]. MGL_1304-specific IgE-induced histamine release from basophils in patients with AD. This suggests the involvement of *M. globosa* in the type I hypersensitivity to sweat observed in some AD individuals. Once triggered, basophil hyper-reactivity to sweat was shown to persist for up to 96 weeks even in successfully treated individuals [94]. 

Proteomic studies of MalaEx, the extracellular nanovesicles of *Malassezia sympodialis*, showed that they are composed of 110 proteins, involving two allergens (Mala s 1 and s 7) [95]. Functional analysis showed that MalaEx can be internalized by keratinocytes and monocytes and trigger TNF-α and IL-4 responses, with the latter significantly more prominent in patients with AD than in the healthy population [91,95]. Moreover, exposition to MalaEx was demonstrated to induce the upregulation of intercellular adhesion molecule-1 on human keratinocytes causing the attraction of immune-competent cells [96].

Interestingly, *Malassezia* spp. seem to release greater amounts of allergens at higher pHs [97]. Therefore, a more alkaline habitat characterizing the skin of patients with AD can result in a higher exposition to *Malassezia* antigens, which contributes to the robust IgE-mediated sensitization against these microbes.

#### 6.1.2. Molecular Base for Malassezia Role in Head and Neck Dermatitis 

The head and neck dermatitis variant of AD poses significant diagnostic and therapeutic challenges. Despite ruling out possible differential diagnoses, such as allergic contact dermatitis and demodicosis, a subset of patients presented persistent lesions in this area [98,99]. Mycobiome dysbiosis was proposed to play a role in this entity based on high rates of sensitization to *Malassezia* antigens and improvements after the administration of antifungals [98,100,101]. Analogous observations have led some authors to attribute dupilumab-induced recalcitrant head and neck dermatitis to *Mallasezia* [99,102,103,104]. Dupilumab is a human monoclonal antibody targeting the IL-4 receptor alpha chain (IL-4Rα) present in both IL-4R complexes, type 1 (IL-4Rα/γc; IL-4 specific) and type 2 (IL-4Rα/IL-13Rα1; IL-4- and IL-13-specific), with the subsequent inhibition of IL-4 and IL-13 function [105]. It could be hypothesized that the downregulation of those two major Th2 cytokines causes the relative predominance of other Th subtypes. This reciprocal regulation was demonstrated between Th2 and Th17 cells in asthma, which implies that there could be similar mechanisms in AD [106]. As mentioned above, *Malassezia* were shown to induce skin inflammation in an IL-17-dependent manner, suggesting a possible role of Th17 in head and neck dermatitis [80]. Furthermore, Th17 cells are known to play a considerable role in selected endotypes and phenotypes of AD, which should encourage epidemiological and interventional studies of head and neck dermatitis in different populations to verify the proposed hypothesis [107]. Nevertheless, regardless of the dominant type of immune reaction, the disrupted epidermal barrier seems to be the key factor in the sensitization to *Malassezia* and its potential role in head and neck dermatitis [75,108].

### 6.2. Other Fungal Species

Mechanistic data on the interactions of other fungal species and the cutaneous immune system are scarce and mostly limited to the *Candida* genus. *C. albicans* was shown to enhance changes in the interaction of keratinocyte-derived small extracellular vesicles and dendritic cells in the AD cytokine milieu [109]. This could be associated with the impaired identification of this pathogen by pattern recognition receptors, corresponding to increased survival on AD skin. In line with this observation, the peripheral blood mononuclear cells of AD patients revealed a decreased proliferation response upon stimulation with *C. albicans* antigens, which reflects the compromised defense against this microbe [110,111]. Simultaneously, in vitro exposition of those cells to *C. albicans* induced the secretion of IL-2, IL-4, IL-5, and IFN-gamma, suggesting the involvement of this species in cutaneous inflammation in AD [112,113]. Furthermore, following contact with *C. albicans*, T cells were shown to produce increased levels of IL-17 [114]. Physiologically, this reaction prevents the development of invasive disease, but in the case of AD, it could also stimulate inflammation through the previously described mechanisms. Lastly, it must be emphasized that the profiles of induced Th1 and Th2 cytokines are dependent on the *Candida* morphotype, i.e., yeast or hyphal [115]. Although Th2 cytokines are upregulated by the hyphal form, the yeast morphotype triggers the Th1 response. This suggests that *Candida* spp. could be implicated in the aggravation of different phases of AD characterized by distinct immunological patterns [48].

Similarly to *Malassezia*, other fungi also trigger immediate hypersensitivity reactions, which can be demonstrated by skin prick tests and the detection of IgE-specific antibodies [116,117,118]. In most studies, IgE sensitization to other fungal species was associated with higher severity of AD [118,119,120]. The production of specific IgE against *C. albicans* antigens was shown to depend on the propensity to induce IFN-gamma by this pathogen [121]. Apart from *Candida* and *Malassezia*, *Cryptococcus diffluens*, *C. liquefaciens*, and *Saccharomyces cerevisiae* were found among the cutaneous fungi causing IgE sensitization in patients with AD [122]. Furthermore, a study implementing ALEX-2 demonstrated increased severity of AD in patients sensitized to allergens of the genera *Alternaria*, *Cladosporium*, *Penicillium*, and *Aspergillus* [118].

As discussed previously, the pan-microbial sensitization involving different species of fungi seems to result primarily from epidermal barrier defects [123]. This is justified by the observation of higher transepidermal water loss in patients exhibiting specific IgE against the three most important pathogens complicating the course of AD, i.e., *S. aureus*, *Malassezia*, and *Candida* [123]. In line with these findings, cross-reactivity between IgE specific to *Candida* species and house dust mites was also demonstrated [124]. 

## 7. Therapeutic Implications

Due to impaired skin barrier function, patients with AD are at increased risk of developing secondary fungal skin infections caused primarily by *Malassezia*, *Candida*, and dermatophytes [125]. The clinical features of these entities are well known and therefore are not discussed. However, selected standard treatment modalities, their effect on the cutaneous mycobiome, and off-label use in particular clinical scenarios are reviewed. Considering that classic antifungal medications can trigger microbial resistance, the search for novel therapeutics has been initiated [126].

### 7.1. Baseline Therapy of Atopic Dermatitis

Baseline therapy for AD can have a beneficial effect on the cutaneous mycobiome. Interventions, such as emollient therapy and irritant removal, improve the epidermal barrier status and reduce the risk of sensitization to environmental factors including the members of the cutaneous mycobiome [127,128]. Emollient use was shown to have a beneficial effect on the cutaneous fungal communities and aid the host-microbe balance on AD skin. In one interventional study, a 12-week emollient application resulted in a significantly decreased richness and increased Shannon diversity on the lesional and nonlesional skin, respectively [129]. Analysis of pre- and post-treatment AD samples revealed distinct microbial clusters at those time points. However, certain components such as olive oil were shown to support *Malassezia* spp. growth and should therefore be avoided [125].

### 7.2. Antifungal Medications

The typical management of fungal skin infections, such as *Malassezia*-associated folliculitis, cutaneous candidiasis, and dermatophytoses, involves topical or oral agents based on the disease type, extent, and severity [125]. Azoles are considered first-line therapy in infections caused by yeasts. Apart from their role in reducing the population of pathogenic fungi, they were reported to inhibit T-cell-dependent IL-4 and IL-5 production [130]. Furthermore, a synergistic fungistatic effect of azoles and tacrolimus was demonstrated in *Malassezia* spp. [131]. This suggests a possible benefit of using these medications in combination therapy, especially in the sensitive skin areas of the head and neck. 

Current European guidelines for the treatment of AD encourage the use of topical or systemic antifungal therapy primarily in patients suffering from the head and neck variant of AD or with demonstrated IgE sensitization to *Malassezia* spp. [132]. As large randomized clinical trials of antifungal use in AD are lacking, proposed treatment regimens can be derived only from small interventional studies, case series, and case reports. One double-blinded placebo-controlled trial of 53 patients randomized to three groups receiving either itraconazole 200 mg/day, itraconazole 400 mg/day, or a placebo for 7 days revealed a significant improvement in the itraconazole-treated individuals [101]. After 14 days, the most significant difference was reported in the group receiving itraconazole 200 mg/day. A retrospective case series showed that treatment with itraconazole 200 mg/day for a mean time of 8.4 months resulted in an improvement of patch-test-negative head and neck dermatitis in 71% of patients [98]. Another report considered adults and adolescents treated with itraconazole 100 mg/day for 1 month and then 100 mg/week for another month as maintenance therapy. Concomitant use of topical steroids and calcineurin inhibitors was allowed. The described treatment regimen resulted in the clearance of head and neck dermatitis in 15/17 (88%) adolescent and 8/14 (57%) adult patients [108].

Favorable results of studies on spontaneous head and neck dermatitis prompted trials evaluating antifungals in the dupilumab-associated variant of this entity. In a prospective evaluation of 25 patients experiencing dupilumab-associated head and neck dermatitis, 13 patients (52%) were treated with a combination corticosteroid/antifungal therapy (ketoconazole or clotrimazole), 10 (40%) with itraconazole 400 mg/day for up to 28 days, and 2 (8%) with topical anti-inflammatory treatment. The response rates were 85%, 70%, and 100%, respectively [102]. However, 80% of patients experienced a recurrence after the cessation of therapy. Another group of 25 patients with dupilumab-associated head and neck dermatitis treated with itraconazole 400 mg/day demonstrated a significant clinical improvement and decrease in *Malassezia*-specific IgE levels, but the discontinuation of antifungal treatment was associated with a rapid recurrence (3–5 days) in 68% of individuals [133]. Lastly, a full or almost full remission of dupilumab-associated head and neck dermatitis following treatment with ketoconazole cream, itraconazole 200 mg/day, or a combination of those therapies for 3 weeks was reported in a case series of 16 patients [99]. 

Considering all presented data, antifungal treatment could alleviate symptoms of head and neck dermatitis in some patients, but the risk of rapid recurrence and the possibility of inducing resistance should prompt careful consideration of the indications before starting the treatment [50,134]. 

### 7.3. Novel Treatments

Fungicidal plant extracts and essential oils were found to show promising in vitro properties against the common cutaneous fungal taxa playing a role in AD. For example, bacillomycin D and dipeptide antibiotic bacilysin extracted from seaweed-associated *Bacillus amyloliquefaciens* were active against several *Malassezia* strains [135]. Furthermore, *Lactiplantibacillus plantarum* derived from green tea was observed to inhibit *C. albicans*, *M. globosa*, and *M. restricta* [136]. The elaboration of emollients containing these preparations could help to control the overgrowth of pathogenic fungal species on the skin of patients with AD. 

Various synthetic antifungals are also under investigation. L-lysine was observed to inhibit the growth of *Malassezia* spp. by targeting homocitrate synthase [137]. Ethyl ester derivatives of free medium- and short-chain fatty acids (such as octanoic acid ethyl ester) hydrolyzed by fungal enzymes were found to generate local and selective activity against *Malassezia*, but not against *Candida* spp. [138]. Esters are a better choice over acids as they lack the unfavorable features of the latter such as the intensive smell and acidity [138]. To inhibit fungal biofilm formation, lipopeptide biosurfactants could be used [139]. Photodynamic therapy with methyl aminolevulinate, 5-aminolevulinic acid, and indole-3-acetic acid leading to the formation of reactive oxygen species also showed activity against *Malassezia* strains in small cohort studies [140,141,142]. Lastly, a *Totiviridae* mycovirus recently isolated from *M. restricta* has been shown to affect the pathogenicity of these fungi [143,144]. Therefore, the prospective use of phage therapy could be further investigated as a therapeutic option for cutaneous mycobiome imbalances.

## 8. Conclusions

Dysbiosis of the cutaneous mycobiome is a characteristic feature of atopic dermatitis. An impaired barrier function facilitates the penetration of fungal antigens, triggering a wide range of immune responses, sensitization to fungal antigens, and subsequent aggravation of skin lesions. The pathogenic role of fungi in atopic dermatitis has been attributed primarily to the genera *Malassezia* and *Candida*. The head and neck variant of atopic dermatitis seems to be the most closely associated with mycobiome dysbiosis due to high rates of detected IgE-specific *Malassezia* antibodies and favorable results following antifungal treatment. Novel treatment options are being developed and could be a successful additive to the routine treatment of atopic dermatitis in the future.

## Figures and Tables

**Table 1:** A summary of the most important mycobiome studies cited in this article, ordered by date of publication. Legend: AD—atopic dermatitis; EC—exclusion criteria; F—female; HC—healthy controls; ITS—internal transcribed spacer; M—male; NR—not reported; PCR-RFLP—polymerase chain reaction—restriction fragment length polymorphism; qPCR—quantitative polymerase chain reaction; y—years.

First Author (Year)	Methodology	Study Population (Number (Sex), Age)		Analyzed Locations	Treatment	Main Findings	Study Limitations
		AD Patients	Healthy Controls				
Choi (2022)	skin swabs ⟶ real-time qPCR	211 (NR), NR;	23 (NR), NR	antecubital fossa	EC: oral or topical antifungals (used within 4 weeks prior to the study)	*C. albicans* and *C. parapsilosis* most prevalent; *Candida* colonization more frequent in AD than in HC	small control group; lack of information about age, sex, and disease severity
Schmid (2022)	skin swabs ⟶ ITS amplicon sequencing	16 (NR), adults;	16 (NR), adults	antecubital fossa, dorsal neck, glabella, vertex	EC: antibiotics or antifungals (within 6 months prior to the study); 14 of 16 AD patients applied topical steroids	*M. restricta* and *M. globosa* most prevalent; decreased *M. restricta* dominance in severe AD patients	small study population; following regular skin care habits; lack of follow-up; various locations difficult to compare; lack of information about age and sex of the study population
Bjerre (2021)	skin swabs ⟶ shotgun metagenomic sequencing	10 (3 M, 7 F), 24–62 y;	5 (2 M, 3 F), 27–63 y	14 locations	EC: antibiotics or probiotics (within 4 weeks prior to the study) 4 AD patients received systemic treatment (not specified)	* Malassezia * less prevalent in AD than HC	small study population, failure in sequencing a substantial number of samples (insufficient biomass); AD patients undergoing systemic treatment included; use of DNA extraction protocol optimized for bacteria; reference databases lack annotations for some organisms (*M. restricta*)
Moosbrugger-Martinz (2021)	skin swabs ⟶ ITS amplicon sequencing	17 (NR), NR;	9 (NR), NR	popliteal fossa, scapular region	NR	*Ascomycota* and *Cladosporium* more frequent in AD than HC	small study population; lack of information about age, sex, and treatment in the study population
Nath (2020)	skin swabs ⟶ shotgun metagenomic sequencing	18 adults (12 M, 6 F), 18–57 y; 16 children (8 M, 8 F), 2–16 y;	54 (NR), 18–57 y	antecubital fossa, neck	EC: antibiotics (within 2 weeks) or any topical cream (within 1 week prior to the study)	*M. globosa* less prevalent in AD than HC	small study population; heterogenous age of the study population
Han (2018)	skin swabs ⟶ ITS amplicon sequencing	10 (NR), NR;	10 (NR), NR	antecubital fossa	EC: oral antifungals, anti-inflammatory drugs, immunomodulators including steroids (within 4 weeks prior to the study); topical antifungals, steroids or calcineurin inhibitors (within 2 weeks prior to the study)	high mycobiome diversity in AD; *M. sloofiae* and *M. dermatis* characteristic of AD	small study population; some isolates might be “transit” microorganisms; lack of information about age and sex of the study population
Chng (2016)	tape stripping, skin swabs, cup scrub samples ⟶ shotgun metagenomic sequencing	19 adults (8 ± 1 M, 12 ± 1 F) *, mean 23.1 y;	15 adults (8 M, 7 F), mean 24.1 y	antecubital fossa, retroauricular crease	NR	*M. restricta* and *M. globosa* most prevalent; *M. sympodialis* and *M. dermatis* characteristic for AD	small study population
Javad (2015)	oral swabs, skin scraps ⟶ culture, D1/D2 26S rRNA sequencing	100 (27 M, 73 F), mean 12.1 ± 11.5 y;	50 (22 M, 28 F), mean 39.9 ± 11.45 y	skin, oral cavity	NR	no significant difference between *Candida* colonization in AD patients and HC	no information about the treatment
Jagielski (2014)	skin swabs ⟶ culture, PCR-RFLP	6 (3 M, 3 F), 22–31 y;	6 (3 M, 3 F), 27–70 y	head, face, chest, back	5 AD patients received topical treatment (emollients/ corticosteroids/ calcineurin inhibitors/ antibiotics) and systemic antihistaminics; 1 AD patient received topical treatment and systemic cyclosporin	*M. sympodialis* most prevalent	use of culture-based approach, possible bias due to treatment
Zhang (2013)	tape stripping ⟶ D1/D2 26S rRNA sequencing	61 (NR), NR;	40 (NR), NR	lesional regions on the face and neck	routine skincare, mild steroid ointment permitted prior to the study	*Cryptococcus albidus* colonization more frequent in AD than HC	lack of information about age and sex of the study population; possible bias due to treatment
Zhang (2011)	tape stripping ⟶ D1/D2 26S rRNA sequencing	9 (NR), NR;	10 (5 M, 5 F), adults	face (lesional site)	medium to strong steroid ointments permitted prior to the study; none of the subjects had received systemic/ topical antibiotics or antifungals	*Malassezia* predominant; *Malassezia* colonization more frequent in AD than HC; mycobiota differs between patients with mild-to-moderate and severe disease	small study population; possible bias due to treatment
Yim (2010)	skin swabs ⟶ culture, PCR-RFLP	60 (30 M, 30 F), 0–30 y	-	scalp, cheek, chest, arm, thigh	EC: systemic glucocorticoids, systemic antifungals, ultraviolet phototherapy (within 2 months prior to the study); topical antifungals (1 month prior to the study); topical corticosteroids (1 week prior to the study); emollients and showers were not allowed on the day of the study	*M. sympodialis* most prevalent; highest colonization on scalp; *M. sympodialis* most common on chest; *M. restricta* most common on scalp and cheeks	heterogenous age of the study population; lack of control group
Tajima (2008)	tape stripping ⟶ nested PCR	36 (24 M, 12 F), 20–64 y;	30 (12 M, 18 F), 20–53 y	face, neck, lesional skin	NR	*M. restricta* and *M. globosa* most prevalent; *Malassezia* colonization more frequent in AD than HC	lack of information about the treatment
Sandström Falk (2005)	contact plates ⟶ culture-based typing (Sabouraud’s agar growth, catalase reaction, Cremophor EL, esculine splitting, Dixon’s agar growth at 38 °C)	124 (42 M, 82 F), adults;	31 (2 M, 29 F), adults	upper back, lesional skin (mainly upper trunk)	NR	*M. sympodialis* most prevalent; *Malassezia* colonization less frequent in AD than HC	lack of information about the treatment
Sugita (2003)	tape stripping ⟶ culture, nested PCR	36 (24 M, 12 F), 20–64 y;	30 (10 M, 20 F), 19–25 y	erythematous lesions on the face and neck	routine skin care, mild steroid ointment permitted prior to the study	*Cryptococcus diffluens* and *C. liquefaciens* colonization more frequent in AD than HC	small study population; possible bias due to treatment
Gupta (2001)	contact plates ⟶ culture-based typing (microscopic observation, catalase reaction, Sabouraud’s agar growth with Tween test; PCR-RFLP of the ITS region to distinguish *M. sympodialis* from *M. furfur* and *M. sloofiae*)	AD: 31 adults (17 M, 14 F), mean 41.3 y;	HC: 20 adults (6 M, 14 F), mean 38.3 y	forehead, arm, trunk, and leg	EC: topical treatment (within 2 weeks prior to the study); oral treatment (within 4 weeks)	*M. sympodialis* most prevalent; *Mallassezia* colonization lower on lesional sites; highest colonization on the forehead	small study population; molecular identification performed only to distinguish between *M. sympodialis* and *M. sloofiae*
Sugita (2001)	tape stripping ⟶ nested PCR	32 (NR), NR;	18 (NR), NR	scalp, back, nape, lesional skin	NR	*M. restricta* and *M. globosa* most prevalent; *Malassezia* colonization more frequent in AD than HC; increased sensitization to *Malassezia* antigens	small study population; lack of information about age, sex, and treatment of the study population
Arzumanyan (2000)	harvesting method not specified ⟶ culture-based typing (morphological, cytological, and physicobiochemical tests; unspecified)	91 (NR), 0.25–36 y	-	mouth edges, cheeks, scalp, face, hands, buttocks	NR	*Candida* colonization more frequent in AD than HC (both lesional and nonlesional skin); highest colonization on the face	lack of control group; lack of information about sex and treatment of the study population; heterogenous age of the study population; unspecified identification methods of the yeast species
Nakabayashi (2000)	skin swabs ⟶ culture-based typing (Sabouraud’s agar growth, Sabouraud’s agar growth with Tween test, catalase reaction, macroscopic and microscopic examination with molecular tests)	17 (8 M, 9 F), 22–41 y;	108 (90 ± 1 M, 18 ± 1 F) *, 22–64 y	scalp, face, trunk (with and without skin lesions)	NR	*Mallassezia* colonization lower on lesional skin; *M. furfur* isolated more frequently from lesional skin than nonlesional skin	small study group; lack of information about the treatment

* a mistake in the population count is present in the article.

## Data Availability

No new data were created or analyzed in this study. Data sharing is not applicable to this article.
